# Supplementary material for: Patient and provider characteristics associated with therapeutic intervention selection in a chiropractic clinical encounter: a cross-sectional analysis of the COAST and O-COAST study data
Source: Chiropr Man Therap. 2023 Sep 21;31:39. doi: 10.1186/s12998-023-00515-y (PMC10512629; doi:10.1186/s12998-023-00515-y)
Supplement: Supplementary file 2 — Supplementary Material 2 [file 12998_2023_515_MOESM2_ESM.docx]

Additional Table 1: Diagnostic groupings

| **Musculoskeletal – Back** | **Musculoskeletal - Neck** | **Musculoskeletal - Head/Jaw** | **Musculoskeletal - Extremity** | **Musculoskeletal – Non Region-Specific** | **Non-musculoskeletal** | **Health maintenance/prevention** |
| --- | --- | --- | --- | --- | --- | --- |
| Thoracic spine problem | Neck problem (includes radiating pain) | Headache/Migraine | Shoulder problem | Osteoarthritis other | Vertigo/dizziness | Health maintenance/prevention |
| Lumbar spine problem |  | Jaw problem | Elbow problem | Muscle problem | Cardiovascular problem |  |
| Pelvis problem |  |  | Arm problem | Musculoskeletal problem other | Disability |  |
| Kyphosis/scoliosis |  |  | Wrist problem | Rehabilitation | Mental health/depression |  |
| Back syndrome with radiating pain |  |  | Hand/finger problem |  | Ear problem |  |
|  |  |  | Ankle problem | Nerve related problem | Feeding problem or irritable infant/child |  |
|  |  |  | Hip problem | Pain, generalised | Other, miscellaneous |  |
|  |  |  | Leg problem |  |  |  |
|  |  |  | Knee problem |  |  |  |
|  |  |  | Foot problem |  |  |  |

Additional Table 2: Therapeutic interventions selected across all diagnostic encounters and for each diagnostic grouping

| **Diagnostic grouping** | **Manipulation** | **Mobilisation** | **Drop piece** | **Instrument adjusting** | **Flexion distraction** | **Blocks** | **Chiropractic system** | **Soft tissue techniques** | **Advice/Education** | **Exercise prescription** | **Modalities** | **Acupuncture** | **Supportive devices** |
| --- | --- | --- | --- | --- | --- | --- | --- | --- | --- | --- | --- | --- | --- |
| All diagnostic encounters N/10731  (%, 95%CI) | 6708 (63, 62-63) | 2105  (20, 19-20) | 2094  (20, 19-20) | 3035  (28, 27-29) | 445  (4, 4-5) | 1269  (12, 11-12) | 431  (4, 4-4) | 6271  (58, 58-59) | 1693  (16, 15-16) | 2841  (26, 26-27) | 2166  (20, 19-21) | 461  (4, 4-5) | 133  (1, 1-1) |
| Musculoskeletal - Back  N/6285  (%, 95%CI) | 4211  (67, 66-68) | 1037  (17, 16-17) | 1575  (25, 24-26) | 1940  (31, 30-32) | 370  (6, 5-7) | 969  (15, 15-16) | 232  (4, 3-4) | 3598  (57, 56-58) | 998  (16, 15-17) | 1511  (24, 23-25) | 1165  (19, 18-20) | 173  (3, 2-3) | 39  (1, 0-1) |
| Musculoskeletal - Neck  N/1391  (%, 95%CI) | 992  (71, 69-74) | 336  (24, 22-26) | 156  (11, 10-13) | 388  (28, 26-30) | 26  (2, 1-3) | 71  (5, 4-6) | 57  (4, 3-5) | 799  (57, 55-60) | 173  (12, 11-14) | 341  (25, 22-27) | 292  (21, 19-23) | 43  (3, 2-4) | 2  (0, 0-1) |
| Musculoskeletal - Head/Jaw  N/293  (%, 95%CI) | 212  (72, 67-77) | 41  (14, 10-18) | 26  (9, 6-13) | 96  (33, 28-38) | 5  (2, 1-4) | 26  (9, 6-13) | 26  (9, 6-13) | 204  (70, 64-75) | 67  (23, 18-28) | 68  (23, 19-28) | 37  (13, 9-17) | 4  (1, 1-3) | 3  (1, 0-3) |
| Musculoskeletal - Extremity  N/985  (%, 95%CI) | 365  (37, 34-40) | 289  (29, 27-32) | 92  (9, 8-11) | 191  (19, 17-22) | 2  (0, 0-1) | 32  (3, 2-5) | 9  (1, 0-2) | 639  (65, 62-68) | 124  (12, 11-15) | 392  (40, 37-43) | 381  (39, 36-42) | 92  (9, 8-11) | 57  (6, 4-7) |
| Musculoskeletal - Non Region-Specific N/1187  (%, 95%CI) | 630  (53, 50-56) | 340  (29, 26-31) | 100  (8, 7-10) | 224  (19, 17-21) | 37  (3, 2-4) | 107  (9, 8-11) | 34  (3, 2-4) | 817  (69, 66-71) | 226  (19, 17-21) | 453  (38, 35-41) | 259  (22, 20-24) | 93  (8, 6-10) | 27  (2, 2-3) |
| Non-musculoskeletal N/315  (%, 95%CI) | 135  (43, 38-48) | 28  (9, 6-13) | 37  (12, 9-16) | 56  (18, 14-22) | 3  (1, 0-3) | 13  (4, 2-7) | 43  (14, 10-18) | 94  (30, 25-35) | 90  (29, 24-34) | 49  (16, 12-20) | 28  (9, 6-13) | 56  (18, 14-22) | 5  (2, 1-4) |
| Health maintenance/ Prevention  N/275  (%, 95%CI) | 163  (59, 53-65) | 34  (12, 9-17) | 108  (39, 34-45) | 140  (51, 45-57) | 2  (1, 0-3) | 51  (19, 14-24) | 30  (11, 8-15) | 120  (44, 38-50) | 15  (5, 3-9) | 27  (10, 7-14) | 4  (1, 1-4) | 0  (0, 0-1) | 0  (0, 0-1) |

Additional Table 3: Association between patient and provider variables and the use of therapeutic interventions across all diagnostic encounters*

| **Diagnostic encounter variable** | **Manipulation** | **Mobilisation** | **Other chiropractic techniques** | **Soft tissue techniques** | **Advice/ Education** | **Exercise prescription** | **Ancillary care** |
| --- | --- | --- | --- | --- | --- | --- | --- |
| Patient sex (Female) | 0.74 (0.65, 0.84) | 1.22 (1.04, 1.43) | 1.10 (0.97, 1.25) | 1.00 (0.88, 1.15) | 1.14 (0.99, 1.32) | 1.01 (0.88, 1.14) | 1.00 (0.87, 1.16) |
| Patient age (Decade) | 0.79 (0.77, 0.82) | 1.19 (1.14, 1.24) | 1.14 (1.10, 1.18) | 1.09 (1.06, 1.13) | 1.02 (0.99, 1.06) | 0.97 (0.94, 1.00) | 1.12 (1.08, 1.17) |
| New patient (Yes) | 0.73 (0.56, 0.95) | 0.96 (0.70, 1.32) | 0.85 (0.64, 1.13) | 0.74 (0.55, 0.99) | 1.50 (1.12, 2.02) | 1.51 (1.16, 1.97) | 1.99 (1.48, 2.68) |
| New complaint (Yes) | 0.82 (0.71, 0.95) | 1.00 (0.84, 1.19) | 0.71 (0.61, 0.82) | 1.08 (0.92, 1.26) | 1.24 (1.06, 1.46) | 1.37 (1.18, 1.58) | 1.96 (1.67, 2.31) |
| Work-related problem (Yes) | 1.62 (1.36, 1.92) | 0.82 (0.66, 1.02) | 0.81 (0.68, 0.96) | 1.35 (1.12, 1.63) | 1.39 (1.16, 1.65) | 1.11 (0.93, 1.32) | 0.94 (0.77, 1.15) |
| Patient BMI (Obese) | 0.69 (0.58, 0.81) | 1.37 (1.11, 1.69) | 1.37 (1.15, 1.62) | 1.03 (0.86, 1.24) | 1.18 (0.98, 1.42) | 0.78 (0.66, 0.93) | 1.18 (0.98, 1.43) |
| Patient BMI (Overweight) | 0.94 (0.81, 1.09) | 1.41 (1.17, 1.70) | 1.05 (0.90, 1.22) | 0.93 (0.79, 1.09) | 0.98 (0.83, 1.16) | 0.89 (0.77, 1.04) | 1.19 (1.01, 1.41) |
| Patient BMI (Underweight) | 0.47 (0.35, 0.63) | 1.24 (0.83, 1.84) | 1.72 (1.26, 2.35) | 0.43 (0.31, 0.59) | 1.00 (0.71, 1.40) | 0.76 (0.56, 1.03) | 0.44 (0.27, 0.71) |
| Patient comorbidities (Yes) | 0.63 (0.54, 0.72) | 1.54 (1.29, 1.84) | 1.53 (1.32, 1.78) | 0.97 (0.83, 1.12) | 1.39 (1.18, 1.62) | 0.85 (0.74, 0.98) | 1.17 (1.00, 1.38) |
| Chiropractor sex (Female) | 0.51 (0.21, 1.22) | 0.71 (0.26, 1.93) | 5.80 (1.71, 19.68) | 1.17 (0.37, 3.74) | 1.25 (0.60, 2.58) | 1.16 (0.49, 2.76) | 0.41 (0.14, 1.18) |
| Chiropractor >5 years (Yes) | 2.48 (0.85, 7.19) | 0.98 (0.28, 3.40) | 2.71 (0.55, 13.3) | 0.48 (0.12, 2.00) | 0.37 (0.16, 0.87) | 0.17 (0.06, 0.44) | 0.50 (0.14, 1.79) |
| Chiropractor weekly visits (25 visits) | 1.01 (0.86, 1.19) | 0.90 (0.74, 1.08) | 1.21 (0.95, 1.53) | 0.76 (0.62, 0.94) | 0.87 (0.76, 1.00) | 0.96 (0.81, 1.13) | 0.96 (0.78, 1.17) |
| Chiropractor teaching (Yes) | 0.41 (0.15, 1.13) | 1.93 (0.56, 6.62) | 1.08 (0.22, 5.33) | 1.02 (0.25, 4.18) | 3.06 (1.29, 7.29) | 2.75 (0.96, 7.89) | 0.91 (0.24, 3.44) |
| Chiropractor country (Canada) | 0.35 (0.16, 0.78) | 2.47 (1.00, 6.12) | 0.29 (0.09, 0.93) | 0.40 (0.14, 1.16) | 0.46 (0.24, 0.89) | 1.66 (0.75, 3.67) | 5.22 (2.10, 12.94) |

* Green highlighted cells show statistical significance with higher association (OR>1); Yellow highlighted cells show statistical significance with lower association (OR<1)

^#^BMI reference category is normal weight

Additional Table 4: Association between patient and provider variables and the use of therapeutic interventions for diagnostic grouping: Musculoskeletal – Back*

| **Diagnostic encounter variable** | **Manipulation** | **Mobilisation** | **Other chiropractic techniques** | **Soft tissue techniques** | **Advice/ Education** | **Exercise prescription** | **Ancillary care** |
| --- | --- | --- | --- | --- | --- | --- | --- |
| Patient sex (Female) | 0.67 (0.57, 0.79) | 1.46 (1.18, 1.81) | 1.19 (1.01, 1.39) | 0.96 (0.81, 1.13) | 1.17 (0.98, 1.40) | 1.05 (0.90, 1.24) | 1.03 (0.85, 1.23) |
| Patient age (Decade) | 0.76 (0.73, 0.79) | 1.23 (1.16, 1.31) | 1.24 (1.18, 1.29) | 1.10 (1.05, 1.15) | 1.03 (0.98, 1.08) | 0.98 (0.94, 1.02) | 1.15 (1.09, 1.21) |
| New patient (Yes) | 0.73 (0.51, 1.05) | 1.20 (0.78, 1.85) | 0.88 (0.61, 1.26) | 0.85 (0.58, 1.26) | 1.52 (1.03, 2.24) | 1.65 (1.17, 2.33) | 1.88 (1.29, 2.76) |
| New complaint (Yes) | 1.03 (0.85, 1.26) | 0.98 (0.77, 1.26) | 0.72 (0.59, 0.87) | 1.10 (0.89, 1.35) | 1.26 (1.02, 1.55) | 1.52 (1.26, 1.85) | 2.13 (1.71, 2.64) |
| Work-related problem (Yes) | 1.65 (1.32, 2.06) | 0.84 (0.62, 1.13) | 0.81 (0.65, 1.01) | 1.30 (1.02, 1.64) | 1.48 (1.20, 1.84) | 1.10 (0.88, 1.36) | 0.89 (0.69, 1.15) |
| Patient BMI (Obese) | 0.62 (0.50, 0.77) | 1.61 (1.21, 2.12) | 1.48 (1.20, 1.84) | 1.09 (0.87, 1.36) | 1.34 (1.06, 1.69) | 0.82 (0.66, 1.01) | 1.28 (1.00, 1.63) |
| Patient BMI (Overweight) | 0.88 (0.73, 1.06) | 1.56 (1.22, 2.01) | 1.23 (1.02, 1.48) | 0.96 (0.79, 1.17) | 1.11 (0.90, 1.37) | 0.95 (0.78, 1.14) | 1.38 (1.11, 1.71) |
| Patient BMI (Underweight) | 0.59 (0.38, 0.90) | 1.50 (0.83, 2.73) | 1.52 (0.99, 2.33) | 0.44 (0.27, 0.70) | 1.02 (0.62, 1.69) | 0.61 (0.38, 0.97) | 0.60 (0.32, 1.14) |
| Patient comorbidities (Yes) | 0.55 (0.46, 0.67) | 1.87 (1.47, 2.36) | 1.74 (1.44, 2.10) | 1.02 (0.84, 1.23) | 1.37 (1.13, 1.66) | 0.92 (0.77, 1.10) | 1.21 (0.99, 1.49) |
| Chiropractor sex (Female) | 0.49 (0.19, 1.24) | 0.59 (0.21, 1.64) | 5.82 (1.70, 19.91) | 1.25 (0.37, 4.24) | 0.93 (0.43, 1.99) | 1.09 (0.45, 2.63) | 0.41 (0.14, 1.23) |
| Chiropractor >5 years (Yes) | 2.77 (0.88, 8.72) | 1.02 (0.28, 3.68) | 2.41 (0.48, 12.06) | 0.68 (0.15, 3.08) | 0.35 (0.14, 0.86) | 0.22 (0.08, 0.61) | 0.46 (0.12, 1.77) |
| Chiropractor weekly visits (25 visits) | 0.98 (0.82, 1.17) | 0.88 (0.72, 1.06) | 1.18 (0.93, 1.49) | 0.74 (0.59, 0.92) | 0.89 (0.77, 1.03) | 0.99 (0.84, 1.16) | 0.94 (0.77, 1.16) |
| Chiropractor teaching (Yes) | 0.42 (0.14, 1.27) | 1.91 (0.53, 6.89) | 1.30 (0.26, 6.55) | 1.26 (0.28, 5.61) | 3.03 (1.21, 7.61) | 2.77 (0.94, 8.20) | 0.94 (0.23, 3.83) |
| Chiropractor country (Canada) | 0.50 (0.21, 1.2) | 2.20 (0.86, 5.62) | 0.34 (0.11, 1.09) | 0.35 (0.12, 1.06) | 0.04 (0.20, 0.79) | 1.57 (0.70, 3.52) | 5.44 (2.11, 14.03) |

* Green highlighted cells show statistical significance with higher association (OR>1); Yellow highlighted cells show statistical significance with lower association (OR<1)

^#^BMI reference category is normal weight

Additional Table 5: Association between patient and provider variables and the use of therapeutic interventions for diagnostic grouping: Musculoskeletal – Neck*

| **Diagnostic encounter variable** | **Manipulation** | **Mobilisation** | **Other chiropractic techniques** | **Soft tissue techniques** | **Advice/ Education** | **Exercise prescription** | **Ancillary care** |
| --- | --- | --- | --- | --- | --- | --- | --- |
| Patient sex (Female) | 0.78 (0.57, 1.07) | 0.80 (0.54, 1.2) | 1.28 (0.93, 1.78) | 1.13 (0.80, 1.59) | 1.22 (0.82, 1.81) | 1.22 (0.87, 1.72) | 0.98 (0.67, 1.44) |
| Patient age (Decade) | 0.78 (0.72, 0.86) | 1.32 (1.17, 1.49) | 1.02 (0.94, 1.12) | 1.03 (0.93, 1.13) | 0.91 (0.82, 1.01) | 0.9 (0.82, 0.99) | 1.07 (0.96, 1.19) |
| New patient (Yes) | 0.69 (0.34, 1.36) | 1.56 (0.70, 3.46) | 1.50 (0.71, 3.20) | 0.38 (0.18, 0.80) | 2.54 (1.18, 5.47) | 1.13 (0.55, 2.35) | 3.65 (1.58, 8.43) |
| New complaint (Yes) | 0.90 (0.61, 1.32) | 1.16 (0.72, 1.86) | 1.01 (0.68, 1.5) | 0.83 (0.55, 1.25) | 1.53 (0.99, 2.38) | 1.29 (0.86, 1.92) | 1.44 (0.89, 2.31) |
| Work-related problem (Yes) | 1.57 (1.00, 2.45) | 0.63 (0.34, 1.17) | 0.60 (0.38, 0.94) | 1.28 (0.77, 2.13) | 1.58 (0.99, 2.52) | 0.86 (0.54, 1.38) | 0.86 (0.48, 1.52) |
| Patient BMI (Obese) | 0.72 (0.47, 1.08) | 1.43 (0.85, 2.39) | 2.06 (1.34, 3.16) | 0.81 (0.51, 1.28) | 0.94 (0.56, 1.57) | 1.04 (0.67, 1.61) | 0.83 (0.50, 1.35) |
| Patient BMI (Overweight) | 1.14 (0.79, 1.66) | 1.04 (0.65, 1.68) | 0.89 (0.61, 1.30) | 0.79 (0.53, 1.18) | 0.83 (0.52, 1.31) | 0.71 (0.47, 1.06) | 0.71 (0.46, 1.12) |
| Patient BMI (Underweight) | 0.22 (0.11, 0.43) | 1.26 (0.44, 3.64) | 5.20 (2.46, 10.97) | 0.36 (0.16, 0.82) | 1.12 (0.47, 2.71) | 1.50 (0.70, 3.21) | 0.32 (0.09, 1.20) |
| Patient comorbidities (Yes) | 0.69 (0.49, 0.97) | 1.54 (1.00, 2.38) | 1.26 (0.88, 1.81) | 1.05 (0.72, 1.54) | 1.64 (1.08, 2.48) | 0.90 (0.63, 1.30) | 1.29 (0.86, 1.93) |
| Chiropractor sex (Female) | 0.44 (0.18, 1.11) | 0.86 (0.25, 2.93) | 3.70 (1.08, 12.68) | 1.47 (0.42, 5.16) | 2.24 (0.95, 5.27) | 1.60 (0.56, 4.51) | 0.92 (0.25, 3.41) |
| Chiropractor >5 years (Yes) | 2.05 (0.69, 6.14) | 0.82 (0.19, 3.53) | 1.37 (0.29, 6.41) | 0.46 (0.10, 2.06) | 0.29 (0.11, 0.78) | 0.23 (0.07, 0.77) | 0.37 (0.08, 1.67) |
| Chiropractor weekly visits (25 visits) | 0.97 (0.82, 1.15) | 0.90 (0.73, 1.12) | 1.10 (0.88, 1.39) | 0.77 (0.62, 0.96) | 0.88 (0.75, 1.02) | 0.96 (0.80, 1.15) | 1.07 (0.85, 1.35) |
| Chiropractor teaching (Yes) | 0.44 (0.16, 1.24) | 1.99 (0.49, 8.13) | 1.54 (0.34, 6.99) | 0.65 (0.16, 2.68) | 2.26 (0.85, 5.96) | 1.17 (0.34, 4.06) | 1.19 (0.25, 5.66) |
| Chiropractor country (Canada) | 0.43 (0.19, 0.99) | 3.83 (1.33, 11.03) | 0.56 (0.18, 1.73) | 1.04 (0.35, 3.13) | 0.58 (0.27, 1.26) | 2.17 (0.86, 5.46) | 4.17 (1.35, 12.9) |

* Green highlighted cells show statistical significance with higher association (OR>1); Yellow highlighted cells show statistical significance with lower association (OR<1)

^#^BMI reference category is normal weight

Additional Table 6: Association between patient and provider variables and the use of therapeutic interventions for diagnostic grouping: Musculoskeletal – Extremity*

| **Diagnostic encounter variable** | **Manipulation** | **Mobilisation** | **Other chiropractic techniques** | **Soft tissue techniques** | **Advice/ Education** | **Exercise prescription** | **Ancillary care** |
| --- | --- | --- | --- | --- | --- | --- | --- |
| Patient sex (Female) | 0.96 (0.65, 1.42) | 0.91 (0.62, 1.36) | 1.07 (0.67, 1.71) | 0.91 (0.61, 1.36) | 1.18 (0.74, 1.90) | 0.72 (0.51, 1.04) | 0.91 (0.61, 1.36) |
| Patient age (Decade) | 0.81 (0.73, 0.90) | 0.99 (0.89, 1.10) | 0.89 (0.78, 1.02) | 0.99 (0.88, 1.10) | 1.06 (0.94, 1.20) | 0.96 (0.87, 1.06) | 1.07 (0.96, 1.19) |
| New patient (Yes) | 0.79 (0.36, 1.74) | 0.67 (0.32, 1.38) | 0.62 (0.21, 1.86) | 0.89 (0.42, 1.89) | 0.56 (0.22, 1.44) | 1.35 (0.70, 2.60) | 0.99 (0.46, 2.14) |
| New complaint (Yes) | 0.86 (0.56, 1.31) | 0.65 (0.43, 0.99) | 0.79 (0.46, 1.37) | 0.84 (0.54, 1.31) | 0.83 (0.50, 1.38) | 0.92 (0.63, 1.33) | 1.84 (1.18, 2.86) |
| Work-related problem (Yes) | 1.63 (0.99, 2.67) | 1.05 (0.64, 1.72) | 1,00 (0.52, 1.93) | 1.33 (0.77, 2.28) | 1.16 (0.66, 2.05) | 0.96 (0.60, 1.52) | 0.73 (0.43, 1.23) |
| Patient BMI (Obese) | 0.96 (0.59, 1.56) | 1.17 (0.70, 1.94) | 1.58 (0.85, 2.93) | 1.63 (0.96, 2.77) | 2.44 (1.34, 4.43) | 0.72 (0.45, 1.15) | 1.24 (0.74, 2.08) |
| Patient BMI (Overweight) | 0.79 (0.50, 1.23) | 1.53 (0.97, 2.42) | 0.76 (0.43, 1.35) | 0.98 (0.61, 1.57) | 1.64 (0.90, 2.98) | 0.93 (0.61, 1.42) | 1.08 (0.68, 1.71) |
| Patient BMI (Underweight) | 0.31 (0.09, 1.02) | 3.85 (1.25, 11.82) | 1.76 (0.45, 6.85) | 0.44 (0.14, 1.38) | 2.88 (0.91, 9.08) | 2.03 (0.75, 5.51) | 0.21 (0.05, 0.83) |
| Patient comorbidities (Yes) | 0.81 (0.52, 1.26) | 0.86 (0.54, 1.36) | 1.17 (0.67, 2.07) | 1.07 (0.68, 1.70) | 1.27 (0.76, 2.13) | 0.80 (0.53, 1.20) | 0.91 (0.58, 1.45) |
| Chiropractor sex (Female) | 0.80 (0.31, 2.07) | 1.08 (0.41, 2.82) | 7.43 (1.93, 28.6) | 1.33 (0.47, 3.78) | 1.10 (0.49, 2.45) | 0.72 (0.32, 1.66) | 0.57 (0.21, 1.52) |
| Chiropractor >5 years (Yes) | 2.20 (0.65, 7.43) | 1.32 (0.39, 4.50) | 2.27 (0.37, 13.94) | 0.32 (0.09, 1.17) | 0.67 (0.26, 1.70) | 0.33 (0.12, 0.87) | 0.87 (0.26, 2.94) |
| Chiropractor weekly visits (25 visits) | 1.04 (0.87, 1.24) | 0.87 (0.73, 1.05) | 1.26 (0.97, 1.63) | 0.79 (0.65, 0.95) | 0.93 (0.80, 1.08) | 0.94 (0.81, 1.10) | 1.00 (0.83, 1.20) |
| Chiropractor teaching (Yes) | 0.35 (0.11, 1.14) | 1.40 (0.44, 4.48) | 1.41 (0.25, 7.91) | 1.12 (0.31, 3.96) | 1.67 (0.64, 4.36) | 3.19 (1.22, 8.39) | 0.47 (0.14, 1.61) |
| Chiropractor country (Canada) | 0.26 (0.12, 0.58) | 1.23 (0.51, 2.94) | 0.21 (0.06, 0.74) | 0.58 (0.23, 1.49) | 0.69 (0.34, 1.40) | 1.18 (0.56, 2.47) | 3.83 (1.64, 8.93) |

* Green highlighted cells show statistical significance with higher association (OR>1); Yellow highlighted cells show statistical significance with lower association (OR<1)

^#^BMI reference category is normal weight

Additional Table 7: Association between patient and provider variables and the use of therapeutic interventions for diagnostic grouping: Musculoskeletal – Non Region-Specific*

| **Diagnostic encounter variable** | **Manipulation** | **Mobilisation** | **Other chiropractic techniques** | **Soft tissue techniques** | **Advice/ Education** | **Exercise prescription** | **Ancillary care** |
| --- | --- | --- | --- | --- | --- | --- | --- |
| Patient sex (Female) | 0.66 (0.48, 0.91) | 1.00 (0.70, 1.44) | 1.08 (0.77, 1.52) | 1.15 (0.80, 1.67) | 1.01 (0.71, 1.44) | 0.85 (0.63, 1.15) | 0.86 (0.59, 1.26) |
| Patient age (Decade) | 0.82 (0.75, 0.89) | 1.24 (1.12, 1.36) | 1.19 (1.09, 1.30) | 1.14 (1.03, 1.27) | 1.07 (0.98, 1.17) | 0.90 (0.83, 0.98) | 0.96 (0.87, 1.06) |
| New patient (Yes) | 0.97 (0.48, 1.95) | 1.14 (0.56, 2.31) | 0.64 (0.31, 1.33) | 0.58 (0.29, 1.17) | 1.25 (0.59, 2.65) | 1.05 (0.56, 1.98) | 2.16 (1.03, 4.56) |
| New complaint (Yes) | 0.53 (0.37, 0.77) | 1.01 (0.67, 1.51) | 0.80 (0.54, 1.19) | 1.26 (0.82, 1.94) | 1.00 (0.67, 1.49) | 1.43 (1.02, 2.02) | 2.03 (1.33, 3.11) |
| Work-related problem (Yes) | 1.95 (1.25, 3.03) | 0.78 (0.48, 1.26) | 0.70 (0.44, 1.12) | 1.02 (0.57, 1.80) | 1.07 (0.68, 1.67) | 1.02 (0.67, 1.55) | 0.62 (0.37, 1.05) |
| Patient BMI (Obese) | 0.57 (0.37, 0.87) | 0.95 (0.59, 1.54) | 1.06 (0.67, 1.67) | 0.87 (0.53, 1.42) | 0.70 (0.44, 1.12) | 0.57 (0.38, 0.87) | 1.05 (0.64, 1.73) |
| Patient BMI (Overweight) | 0.69 (0.46, 1.04) | 1.15 (0.73, 1.80) | 0.72 (0.47, 1.10) | 1.32 (0.82, 2.11) | 0.81 (0.52, 1.25) | 1.08 (0.74, 1.56) | 1.10 (0.69, 1.75) |
| Patient BMI (Underweight) | 0.34 (0.15, 0.75) | 0.60 (0.24, 1.52) | 1.65 (0.75, 3.65) | 0.58 (0.25, 1.37) | 0.27 (0.10, 0.73) | 0.85 (0.42, 1.72) | 0.51 (0.15, 1.75) |
| Patient comorbidities (Yes) | 0.58 (0.40, 0.86) | 2.15 (1.42, 3.24) | 1.93 (1.26, 2.95) | 0.97 (0.64, 1.47) | 1.45 (0.96, 2.18) | 0.69 (0.48, 0.98) | 1.33 (0.85, 2.08) |
| Chiropractor sex (Female) | 0.84 (0.36, 1.93) | 0.48 (0.18, 1.29) | 4.26 (1.58, 11.52) | 1.14 (0.40, 3.19) | 0.77 (0.34, 1.75) | 1.05 (0.51, 2.18) | 0.40 (0.14, 1.14) |
| Chiropractor >5 years (Yes) | 1.32 (0.48, 3.62) | 2.28 (0.68, 7.68) | 1.64 (0.45, 6.05) | 0.66 (0.19, 2.30) | 0.69 (0.27, 1.77) | 0.30 (0.13, 0.70) | 0.87 (0.25, 3.02) |
| Chiropractor weekly visits (25 visits) | 0.90 (0.77, 1.06) | 1.04 (0.87, 1.25) | 1.20 (0.98, 1.47) | 0.74 (0.61, 0.90) | 0.98 (0.84, 1.14) | 0.98 (0.85, 1.13) | 1.09 (0.89, 1.34) |
| Chiropractor teaching (Yes) | 0.87 (0.32, 2.35) | 3.76 (1.23, 11.49) | 0.84 (0.23, 3.10) | 2.20 (0.63, 7.7) | 3.27 (1.31, 8.16) | 3.46 (1.48, 8.10) | 1.12 (0.30, 4.18) |
| Chiropractor country (Canada) | 0.27 (0.13, 0.55) | 2.40 (1.01, 5.73) | 0.27 (0.10, 0.70) | 0.32 (0.13, 0.82) | 0.50 (0.24, 1.06) | 1.30 (0.66, 2.55) | 6.18 (2.57, 14.88) |

* Green highlighted cells show statistical significance with higher association (OR>1); Yellow highlighted cells show statistical significance with lower association (OR<1)

^#^BMI reference category is normal weight
