# Supplementary material for: Patient and provider characteristics associated with therapeutic intervention selection in a chiropractic clinical encounter: a cross-sectional analysis of the COAST and O-COAST study data
Source: Chiropr Man Therap. 2023 Sep 21;31:39. doi: 10.1186/s12998-023-00515-y (PMC10512629; doi:10.1186/s12998-023-00515-y)
Supplement: Supplementary file 3 — Supplementary Material 3 [file 12998_2023_515_MOESM3_ESM.docx]

**Patient and provider characteristics associated with therapeutic intervention selection in a chiropractic clinical encounter: a cross-sectional analysis of the COAST and O-COAST study data**

Hazel J Jenkins: [hazel.jenkins@mq.edu.au](mailto:hazel.jenkins@mq.edu.au); Department of Chiropractic, Level 2, 75 Talavera Rd, Macquarie University, Australia, 2109; Corresponding author

Aron Downie: [aron.downie@mq.edu.au](mailto:aron.downie@mq.edu.au); Department of Chiropractic, Macquarie University, Australia

Jessica J Wong: [jessica.wong@ontariotechu.ca](mailto:jessica.wong@ontariotechu.ca); Institute for Disability and Rehabilitation Research, Ontario Tech University, Canada

James J Young: [james.young2@uhn.ca](mailto:james.young2@uhn.ca); Schroeder Arthritis Institute, Krembil Research Institute, University Health Network, Canada; Center for Muscle and Joint Health, Department of Sports Science and Clinical Biomechanics, University of Southern Denmark, Denmark

Eric J Roseen: [ericjroseen@gmail.com](mailto:ericjroseen@gmail.com); Section of General Internal Medicine, Department of Medicine, Boston University Chobanian & Avedision School of Medicine and Boston Medical Center, USA

Casper Glissmann Nim: [casper.nim@rsyd.dk](mailto:casper.nim@rsyd.dk); Center for Muscle and Joint Health, Department of Sports Science and Clinical Biomechanics, University of Southern Denmark, Denmark; Department of Regional Health Research, University of Southern Denmark, Denmark; Spine Centre of Southern Denmark, University of Southern Denmark, Denmark

David McNaughton: [david.mcnaughton@mq.edu.au](mailto:david.mcnaughton@mq.edu.au); Department of Chiropractic, Macquarie University, Australia

Cecilie K Øveras: cecilie.overas@ntnu.no; Department of Public Health and Nursing, Norwegian University of Science and Technology (NTNU), Norway

Jan Hartvigsen: [jhartvigsen@health.sdu.dk](mailto:jhartvigsen@health.sdu.dk); Center for Muscle and Joint Health, Department of Sports Science and Clinical Biomechanics, University of Southern Denmark, Denmark; Chiropractic Knowledge Hub, Denmark

Silvano Mior: [smior@cmcc.ca](mailto:smior@cmcc.ca); Institute for Disability and Rehabilitation Research, Ontario Tech University, Canada; Department of Research and Innovation, Canadian Memorial Chiropractic College, Canada

Simon D French: [simon.french@mq.edu.au](mailto:simon.french@mq.edu.au); Department of Chiropractic, Macquarie University, Australia

**Abstract**

Background: Chiropractors use a variety of therapeutic interventions in clinical practice. How the selection of interventions differs across musculoskeletal regions or with different patient and provider characteristics is currently unclear. This study aimed to describe how frequently different interventions are used for patients presenting for chiropractic care, and patient and provider characteristics associated with intervention selection.

Methods: Data were obtained from the Chiropractic Observation and Analysis STudy (COAST) and Ontario (O-COAST) studies: practice-based, cross-sectional studies in Victoria, Australia (2010-2012) and Ontario, Canada (2014-2015). Chiropractors recorded data on patient diagnosis and intervention selection from up to 100 consecutive patient visits. The frequency of interventions selected overall and for each diagnostic category (e.g., different musculoskeletal regions) were descriptively analysed. Univariable multi-level logistic regression (provider and patient as grouping factors), stratified by diagnostic category, was used to assess the association between patient/provider variables and intervention selection.

Results: Ninety-four chiropractors, representative of chiropractors in Victoria and Ontario for age, sex, and years in practice, participated. Data were collected on 7,966 patient visits (6419 unique patients), including 10,731 individual diagnoses (mean age: 43.7 (SD: 20.7), 57.8% female). Differences in patient characteristics and intervention selection were observed between chiropractors practicing in Australia and Canada. Overall, manipulation was the most common intervention, selected in 63% (95%CI:62-63) of encounters. However, for musculoskeletal conditions presenting in the extremities only, soft tissue therapies were more commonly used (65%, 95%CI:62-68). Manipulation was less likely to be performed if the patient was female (OR:0.74, 95%CI:0.65-0.84), older (OR:0.79, 95%CI:0.77-0.82), presenting for an initial visit (OR:0.73, 95%CI:0.56-0.95) or new complaint (OR:0.82, 95%CI:0.71-0.95), had one or more comorbidities (OR:0.63, 95%CI:0.54-0.72), or was underweight (OR:0.47, 95%CI:0.35-0.63), or obese (OR:0.69, 95%CI:0.58-0.81). Chiropractors with more than five years clinical experience were less likely to provide advice/education (OR:0.37, 95%CI:0.16-0.87) and exercises (OR:0.17, 95%CI:0.06-0.44).

Conclusion: In more than 10,000 diagnostic encounters, manipulation was the most common therapeutic intervention for spine-related problems, whereas soft tissue therapies were more common for extremity problems. Different patient and provider characteristics were associated with intervention selection. These data may be used to support further research on appropriate selection of interventions for common musculoskeletal complaints.

**Keywords:** Chiropractic; Therapeutic intervention; Musculoskeletal disorders; Patient characteristics; Provider characteristics

**Background**

As defined by the World Health Organisation, chiropractic is a ‘health care profession concerned with the diagnosis, treatment and prevention of disorders of the neuromusculoskeletal system and the effects of these disorders on general health’ [1]. There are more than 100,000 chiropractors worldwide, with representation in 90 countries [2]. Chiropractors provide management for a range of disorders that affect the musculoskeletal system, with back and neck pain or extremity conditions being the most commonly reported reasons for attending chiropractic care [3]. Rarer reasons for attending chiropractic care are wellness/maintenance care and non-musculoskeletal conditions [3].

Chiropractors use a variety of therapeutic interventions in the management of musculoskeletal disorders [1], including joint manipulation/mobilisation, soft tissue techniques, advice/education, exercise prescription, and other therapies (e.g., dry needling, laser, shockwave) [3, 4]. Joint manipulation is the most common therapeutic intervention to be used by chiropractors, used in approximately 80% of clinical encounters, while other interventions are used in approximately 35% of encounters or less [3]. Why a chiropractor decides to use manipulation or a different intervention in a particular clinical encounter has yet to be explored in the literature.

The selection of therapeutic interventions for a particular patient should include consideration of current evidence, healthcare provider experience, and patient preference [5]. Clinical practice guidelines summarise current evidence; however, interventions are only endorsed in general terms (e.g., manipulation) for common conditions (e.g., low back pain) rather than providing advice specific to individual clinical encounters [6]. Healthcare provider knowledge, beliefs, and experiences impact the selection of interventions, and decisions may vary depending on different characteristics of the clinical encounter. Further, the type of presenting condition has been shown to impact intervention selection. For example, chiropractors report being less likely to use spinal manipulation for cervical spine conditions with, rather than without, neurological involvement [7]. However, little is known about how patient characteristics (e.g., age, initial visit, work-related complaint) and provider characteristics (e.g., years in practice, average number of patient visits per week) are associated with the selection of specific therapeutic interventions.

The Chiropractic Observation and Analysis STudy (COAST) [8] and the Ontario Chiropractic Observation and Analysis STudy (O-COAST) [9] are cross-sectional studies that collected data from nearly 8,000 patient visits. Data collected included patient and chiropractor characteristics, clinical diagnosis, and therapeutic intervention selection. These data provide an opportunity to investigate the frequency of intervention use and associations between clinical encounter characteristics and intervention selection. Therefore, using data from the COAST and O-COAST studies, the aims of this study were to:

1. Describe the frequency of use of different therapeutic interventions in patients presenting for chiropractic care in Victoria, Australia (COAST) and Ontario, Canada (O-COAST), across all encounters and stratified by diagnostic groupings; and
2. Explore the association between patient and provider characteristics and the selection of therapeutic intervention, across all encounters and stratified by diagnostic groupings.

**Methods**

We conducted a cross-sectional study using data obtained from COAST [8] and O-COAST [9], which are practice-based, cross-sectional studies conducted in Victoria, Australia (2010-2012) and Ontario, Canada (2014-2015), respectively. Both COAST and O-COAST used similar methods for recruitment and data collection. Ethics approval for COAST was provided by the University of Melbourne Human Research Ethics Committee (HREC: 0931651) and for O-COAST by the Canadian Memorial Chiropractic College (REB: 1404X03) and Queen’s University (REB: 6012853) ethics boards. This paper has been reported in accordance with the STrengthening the Reporting of OBservational studies in Epidemiology (STROBE) statement [10].

COAST and O-COAST data

Study design, chiropractor recruitment, data collection, and coding of free-text data for the COAST and O-COAST studies have been previously described [8, 9, 11]. Similar study processes were used for COAST and O-COAST, except for minor changes from free-text to tick-box options for two items on the data collection form. Fifty-two chiropractors were recruited for COAST, including 14 females (27%), mean age of 42 years (SD 9.3), mean years in practice of 16 years (SD 8.5), mean patient visits per week of 86 visits (SD 48.2), and 8 involved in teaching (15%). Forty-two chiropractors were recruited for O-COAST, including 14 females (33%), mean age of 44 years (SD 11.4), mean years in practice of 15 years (SD 11.0), mean patient visits per week of 100 visits (SD 78.1), and 7 involved in teaching (17%). The 52 chiropractors participating in COAST and the 42 chiropractors participating in O-COAST were representative of chiropractors in Victoria [8] and Ontario [12] in terms of age, sex, and years in practice.

Participating chiropractors were asked to record anonymous patient encounter data from up to 100 consecutive patient visits across a 4-week period (this could include multiple visits from the same patient over the 4-week period). All patient visits were eligible for inclusion. For each patient visit, chiropractors used a form with free-text or tick-box options [8, 9] to record patient characteristics (e.g., age, sex, height, weight, co-morbidities, new patient with the chiropractor) and up to three individual diagnoses that were addressed during that patient visit (each defined as a diagnostic encounter). For each diagnostic encounter, the chiropractor recorded the therapeutic interventions used, whether it was a new or old complaint, and whether the complaint was related to work. Chiropractors completed the data collection form during the consult with the patient, or immediately afterwards. All patient encounters collected in COAST and O-COAST were included in this analysis.

Free-text data for diagnostic encounters and therapeutic interventions were coded by trained coders using the International Classification of Primary Care, 2nd edition (ICPC-2), using the Australian ICPC-2 PLUS general practice terminology [13] and the ICPC-2 PLUS Chiro terminology [11].

Outcome: Use of therapeutic intervention

The primary outcome was the use of different therapeutic interventions for each diagnostic encounter. Therapeutic interventions were categorised into 13 categories based on the type of intervention (e.g., manipulation, mobilisation, advice/education), as defined in Table 1. The outcome of selecting each intervention (e.g., manipulation) for each diagnostic encounter was categorised as yes or no.

Diagnostic groupings

Outcomes were assessed across all diagnostic encounters and stratified by diagnostic groupings. Each diagnostic encounter was categorised into one of seven diagnostic groupings, based on the type and region of complaint: Musculoskeletal – Back (thoracic spine, lumbar spine, pelvis); Musculoskeletal – Neck (cervical spine); Musculoskeletal – Head/Jaw (headache/migraine, temporomandibular joint); Musculoskeletal – Extremity (upper or lower extremity); Musculoskeletal – Non region-specific (e.g., arthritis, nerve problem, musculoskeletal problem without a defined location); Non-musculoskeletal (complaints outside the musculoskeletal system e.g., vertigo, mental health); and Health maintenance/Preventative care (Additional Table 1, in Additional File 1).

Independent variables: Patient and provider characteristics

Patient and provider characteristics available in the COAST and O-COAST datasets were discussed amongst the authors and selected for inclusion in the analysis if hypothesized to be associated with the selection of therapeutic interventions. Discussion among the author team was based upon their: (i) clinical experience as chiropractors; (ii) education experience in chiropractic teaching institutions; and (iii) reference to current literature. Considerations discussed included: (i) patient and provider characteristics commonly described in the literature when describing chiropractic practice or the use of treatments (e.g., age, sex, work-related complaint, BMI) [3, 14]; (ii) the likelihood of a patient characteristic to change the level of benefit or risk of adverse events associated with an intervention (e.g., older age or presence of comorbidities and increased risk of adverse events with manipulation) [15]; and (iii) the potential of a provider characteristic to be associated with provider experience or decision-making (e.g., years in practice, involvement in teaching) [16, 17]. We included the following patient characteristics: age (calculated as a continuous variable, per decade of age); sex (male/female); presence of at least one comorbidity (yes/no); body mass index (BMI), categorised as underweight (<18.5), normal weight (18.5-<25; reference category), overweight (25-<30), and obese (≥30) [18]; initial presentation of a patient to the provider (yes/no); new complaint for the patient (yes/no); and whether the patient presented with a work-related complaint (yes/no). Provider characteristics included: sex (male/female); >5 years in clinical practice (yes/no); average number of patient visits per week (calculated as a continuous variable, per 25 patient visits); involved in teaching (yes/no); and country of practice (Australia/Canada). More than five years in clinical practice was selected as a cut-point to explore whether less clinical experience, and less time since entry-level clinical training, are associated with different treatment selections. Reducing the cut-point further (e.g., two or three years) reduced the number of patients in the lower clinical experience group, and would not allow for meaningful assessment. No imputation was performed for missing data.

Data analysis

*Aim 1: Frequency of use of therapeutic interventions*

Counts of use of each therapeutic intervention were presented and proportions of use, with Wald 95% confidence intervals (95%CI), across all diagnostic encounters calculated. Results were presented with rounding to the nearest whole number. Frequency of use was presented across all diagnostic encounters and stratified for each diagnostic grouping.

*Aim 2: Patient and provider characteristics associated with therapeutic intervention selection*

Generalised mixed models were used to perform univariable and multilevel logistic regression analyses to assess the association between each of the previously stated patient and provider characteristics as independent variables and use of the therapeutic intervention (yes/no) as the dependent variable. Multilevel models were used to account for clustering of diagnostic encounters (level 1) within individual patients (level 2) and providers (level 3). Due to low counts in some intervention categories (used in less than 10% of the diagnostic encounters), therapeutic interventions were further categorised as ‘Other chiropractic techniques’: techniques addressing joint movement using additional equipment/systems (drop-piece, instrument adjusting, flexion-distraction, blocks, or chiropractic systems); or ‘Ancillary therapies’: techniques addressing soft tissues using additional equipment (modalities, acupuncture, or supportive devices). Separate models were created for each therapeutic intervention category across all diagnostic encounters and stratified by four of the diagnostic groupings: musculoskeletal-back, musculoskeletal-neck, musculoskeletal-extremity, and musculoskeletal-non region-specific. The remaining diagnostic groupings had less than 350 diagnostic encounters included in each, an insufficient sample size to perform the analysis. Estimates are presented as odds ratios (OR) with Wald 95%CI. All analyses were performed in IBM SPSS Statistics, Version 27.

**Results**

Sample characteristics

Data were collected on 6,419 unique patients attending 7,966 patient visits (median 1 patient visit; range 1-10), which included 10,731 diagnostic encounters (median 1 diagnostic encounter; range 1-3). Missing data varied from 2.70% (patient sex) to 17.47% (work-related presentation); however, missingness did not appear to be random. Missing data were more common for some providers, often with missing data across multiple variables from the same encounter. Variables with the highest proportion of missing data (new patient, new complaint, work-related, all >15%) were more likely to be left blank if a ‘no’ response was intended.

The characteristics of the patients and providers and the diagnostic groupings selected across all diagnostic encounters are presented in Table 2. Across all diagnostic encounters, mean patient age was 43.7 years (SD: 20.7) and 58% of patients were female. Overall, only 6.5% of diagnostic encounters were patients who were presenting for an initial visit with the provider, but 33.4% of diagnostic encounters were for a new complaint. Patients at 28.3% of the diagnostic encounters had at least one comorbidity, and more than half of the diagnostic encounters were for overweight (34.4%) or obese (23.7%) patients. Providers generally had more than five years clinical experience (87.2%) and were more likely to be male (69.4%). The most common diagnostic grouping to present was Musculoskeletal – Back (58.6%). Very few diagnostic encounters were for non-musculoskeletal complaints (Non-musculoskeletal, 2.9%; Health maintenance/Preventative care, 2.6%). Diagnostic encounter characteristics were largely similar between Australia and Canada, with a difference of 5% or less for most characteristics. Diagnostic encounters from Australia were more likely to be for new and work-related complaints and less like to be for musculoskeletal complaints in the extremities. Australian providers were more likely than Canadian providers to have been in practice for five or more years but tended to see fewer patients per week on average.

*Aim 1: Frequency of use of therapeutic interventions*

Across all diagnostic encounters, manipulation was the most common therapeutic intervention used (63%, 95%CI: 62-63), followed by soft tissue techniques (58%, 95%CI: 58-59) (Figure 1 and Additional Table 2, in Additional File 1). Flexion distraction, chiropractic systems, acupuncture, and supportive devices were all used in less than 5% of diagnostic encounters. Similar use of therapeutic interventions were observed across the different diagnostic groupings except for Musculoskeletal – Extremity, where soft tissue techniques (65%, 95%CI: 62-68), exercise prescription (40%, 95%CI: 37-43), and modalities (39%, 95%CI: 36-42) were the most common interventions used; and Musculoskeletal – Non region-specific, where soft tissue techniques (69%, 95%CI: 66-71) was the most common intervention used.

Therapeutic intervention selection across all diagnostic encounters showed differences between encounters conducted in Australia (COAST data) and Canada (O-COAST data) for all interventions except instrument adjusting, flexion distraction, and supportive devices (Figure 2). Australian chiropractors were more likely to select manipulation, drop piece, blocks, soft tissue techniques, and advice/education, and were less likely to select mobilisation, chiropractic systems, exercise prescription, modalities, or acupuncture.

*Aim 2: Patient and provider characteristics associated with therapeutic intervention selection*

Associations across all diagnostic encounters

Across all diagnostic encounters, the odds of patients receiving manipulation (versus not receiving manipulation) were lower if they were female (OR: 0.74, 95%CI: 0.65-0.84), older (per decade; OR: 0.79, 95%CI: 0.77-0.82), presenting for an initial visit to the provider (OR: 0.73, 95%CI: 0.56-0.95), presenting with a new complaint (OR: 0.82, 95%CI: 0.71-0.95), had one or more comorbidities (OR: 0.63, 95%CI: 0.54-0.72), or were underweight (BMI<18.5; OR: 0.47, 95%CI: 0.35-0.63) or obese (BMI>30; OR: 0.69, 95%CI: 0.58-0.81) (Figures 3 and 4 and Additional Table 3, in Additional File 1). The odds of receiving manipulation were higher if the presentation was for a work-related complaint (OR: 1.62, 95%CI: 1.36-1.92). In contrast, older patients (per decade) had higher odds of receiving mobilisations (OR: 1.19, 95%CI: 1.14-1.24), other chiropractic techniques (OR: 1.14, 95%CI: 1.10-1.18), soft tissue techniques (OR: 1.09, 95%CI: 1.06-1.13), and ancillary care (OR: 1.12, 95%CI: 1.08-1.17). Patients presenting for an initial visit to the provider had higher odds of receiving advice/education (OR: 1.50, 95%CI: 1.12-2.02), exercise prescription (OR: 1.51, 95%CI: 1.16-1.97), or ancillary therapies (OR: 1.99, 95%CI: 1.48-2.68), but lower odds of receiving soft tissue techniques (OR: 0.74, 95%CI: 0.55-0.99), with similar associations for patients with a new complaint. Finally, patients with one or more comorbidities had higher odds of receiving mobilisation (OR: 1.54, 95%CI: 1.29-1.84), other chiropractic techniques (OR: 1.53, 95%CI: 1.32-1.78), and advice/education (OR: 1.39, 95%CI: 1.18-1.62), and had lower odds of receiving an exercise prescription (OR: 0.85, 95%CI: 0.74-0.98). Similar associations were observed for patients who were underweight or obese when compared with a normal BMI range, except that those who were underweight also had lower odds of receiving soft tissue techniques (OR: 0.43, 95%CI: 0.31-0.59).

Different provider variables were also associated with therapeutic intervention selection. Female providers had higher odds of performing other chiropractic techniques (OR: 5.80, 95%CI: 1.71-19.68); providers in practice for more than five years had lower odds of providing advice/education (OR: 0.46, 95%CI: 0.24-0.89) or exercise prescription (OR: 0.17, 95%CI: 0.06-0.44); providers seeing a higher number of average patient visits (per 25 visits) had lower odds of using soft tissue techniques (OR: 0.76, 95%CI: 0.62-0.94); chiropractors involved in teaching had higher odds of providing advice/education (OR: 3.06, 95%CI: 1.29-7.29); and Canadian providers had lower odds of using manipulation (OR: 0.35, 95%CI: 0.16-0.78), other chiropractic techniques (OR: 0.29, 95%CI: 0.09-0.93), or providing advice/education (OR: 0.46, 95%CI: 0.24-0.89), but higher odds of using ancillary therapies (OR: 5.22, 95%CI: 2.10-12.94).

Associations for each diagnostic grouping

Similar patterns in associations were generally observed across the individual diagnostic groupings (Additional Tables 4-7, in Additional File 1) as seen across all diagnostic encounters. However, the fewer diagnostic encounters available in each diagnostic grouping resulted in wider confidence intervals. Differences in associations were observed in the Musculoskeletal – Neck diagnostic grouping, where older patients (per decade) had lower odds of receiving an exercise prescription (OR: 0.9, 95%CI: 0.82-0.99). In the Musculoskeletal – Extremity grouping, patients with a new complaint and underweight patients had lower odds of receiving mobilisation (OR: 0.65, 95%CI: 0.43-0.99; OR: 3.85, 95%CI: 1.25-11.82 respectively) and obese patients had higher odds of receiving advice/education (OR: 2.44, 95%CI: 1.34-4.43). Finally, in the Musculoskeletal – Non region-specific diagnostic grouping, underweight patients had lower odds of receiving advice/education (OR: 0.27, 95%CI: 0.10-0.73). Providers involved in teaching had higher odds of performing mobilisations (OR: 3.76, 95%CI: 1.23-11.49) and providing an exercise prescription (OR: 3.46, 95%CI: 1.48-8.10), and Canadian providers had lower odds of performing soft tissue techniques (OR: 0.32, 95%CI: 0.13-0.82).

**Discussion**

*Key findings*

Across more than 10,000 diagnostic encounters in chiropractic practice in Australia and Canada, manipulation and soft tissue techniques were the most common therapeutic interventions to be selected, used in more than half of the encounters. Similar frequencies in intervention selection were observed across individual diagnostic groupings, except for extremity and non region-specific musculoskeletal conditions, where manipulation was used in less than half of diagnostic encounters.

Across all conditions, patients were less likely to receive manipulation if they were female, of older age, presenting to the chiropractor for the first time, had a new complaint, had one or more comorbidities, or were underweight or obese. Conversely, mobilisations, advice/education, other chiropractic techniques, and ancillary care were more likely to be used for patients presenting with these characteristics. Chiropractors who had been in practice for more than five years were less likely to report using advice/education or prescribe exercises.

*Comparison to previous literature*

A 2017 scoping review [3] found 34 studies, including the COAST study [8], that assessed the types of therapeutic interventions selected by chiropractors. Similar to this study, joint manipulation and soft tissue techniques were the most frequent interventions provided in 79% and 35% of treatments respectively [3]. However, patient education and the use of supportive devices were more commonly reported in the scoping review compared to this study at 31% and 13% respectively [3]. Differences in reporting may be related to the method of data collection, where advice/education and supportive devices were coded from free-text entries in the COAST and O-COAST studies and may not have been consistently reported. However, use of exercise prescription, which was also coded from free-text in the COAST and O-COAST studies, was consistent with the 26% usage reported in 14 studies in the scoping review [3]. A survey of Australian chiropractors reported on therapeutic intervention selection for specific spinal conditions [7]. Similar to this study, they reported that manipulation and soft tissue techniques were the most commonly selected interventions overall and that drop-piece and flexion-distraction were more commonly used in conditions in the back compared to those in the neck. However, the survey reported increased use of instrument adjusting in the neck compared to the back, which was not observed in this study [7]. In particular, the survey reported instrument adjusting as the preferred therapeutic intervention for neck conditions with nerve involvement [7]. In the current study, due to the broad diagnostic categories used, it was not possible to separate the neck conditions with nerve involvement from those without. Therefore, it is unknown whether the presence or absence of nerve involvement was associated with intervention selection.

*Strengths and limitations*

Strengths of this study include the large data set, incorporating over 10,000 diagnostic encounters from Victoria, Australia and Ontario, Canada. The data were systematically collected using piloted data collection forms from up to 100 consecutive patient visits per participating chiropractor. Data were collected prospectively, with the chiropractor completing the data collection form during the consult with the patient. Data on use of therapeutic interventions were collected using a systematic tick-box approach for most interventions assessed; however, free-text data entry for other interventions (e.g., advice/education, exercise prescription) may have introduced measurement error with possible under-reporting of use. Patient diagnosis was recorded using free-text due to the wide variety of potential diagnoses. However, potential variations in interpretation of free-text data entries between researchers categorising the data were limited by using an established coding system [13], with appropriate modifications for chiropractic-specific terminology [11]. A cross-sectional study design was used, with the independent and outcome variables collected during the same patient visit. Cross-sectional study designs are typically limited in their ability to capture the temporality of an association, i.e., it is unknown whether the independent variable preceded the outcome variable or not. However, in this study the outcome variable (choice of treatment) is related to a clinical decision made by the chiropractor, where they would already be aware of the collected independent variables (e.g., patient age, sex, work-related complaint). Therefore, it is likely that temporal associations between the independent and outcome variables are present.

A random selection of chiropractors was approached to participate in the COAST and O-COAST studies; however, participant response rates of 33% [8] and 35% [9], respectively, may have introduced selection bias. Participating chiropractors were broadly representative of chiropractors in Victoria and Ontario in terms of age, sex, and years in practice [8, 12]. A higher percentage of chiropractors in COAST and O-COAST were involved in teaching compared to Australian registered chiropractors, where approximately 1% are involved in teaching [19]. Data collected in Australia and Canada were combined in this analysis to enable exploration of differences between regions. While the study processes and data collection methods were similar, two therapeutic interventions (modalities, acupuncture) were collected with free-text entry in COAST and tick-box entry in O-COAST, potentially explaining some of the differences in intervention use for modalities and acupuncture observed between the two cohorts.

*Implications*

This study summarises the therapeutic interventions selected for different diagnostic presentations by chiropractors in Victoria, Australia and Ontario, Canada, and describes patient and provider characteristics that may be associated with intervention selection. Clinical practice guidelines for the treatment of musculoskeletal conditions commonly recommend the use of advice/education, exercise prescription, and joint manipulation/mobilisation [6, 20-22]. While joint manipulation/mobilisation were commonly used in this study, provision of advice/education and exercises were less frequently reported and may indicate inconsistencies with clinical practice guideline recommendations that need to be further studied and addressed. Measurement of the use of advice/education and exercise prescription may have been affected by the data collection method (free-text entry). Future research in this area is warranted to understand how frequently chiropractors use advice/education and exercise prescription in clinical practice, assess alignment with current guideline recommendations, and understand why some chiropractors do not prescribe exercises or provide advice. Of note, the provision of advice/education and exercises was associated with the clinical experience of the provider, whereby those with less clinical experience were more likely to provide these interventions. The differences in intervention selection observed between more recent and more experienced clinicians may reflect changing education content for newer graduates and potential need for further implementation strategies to promote the uptake of clinical guidelines with more experienced chiropractors. The COAST and O-COAST data were collected between 2010-2015 and it is unknown whether greater alignment between guideline recommendations and intervention selection (such as exercise prescription and advice) may have occurred since then. However, challenges related to the delivery of high-quality education/advice in clinical practice and the need for the development of tools or training continue to be identified [23] and it is likely that implementation challenges still remain in this area.

This study identified differences in therapeutic intervention selection between chiropractors in Australia and Canada. These differences may be related to regional differences in culture, education, healthcare systems, or the different time-periods of data collection. However, it is possible that there are differences in clinical practice behaviour between geographic regions which should be considered in recommendations for future research to inform clinical practice. Findings suggest that several patient and provider characteristics were associated with the selection of therapeutic interventions. In particular, a number of patient characteristics (e.g., older age, one or more comorbidities) were associated with lower odds of receiving joint manipulation. While the reasons for chiropractors being less likely to perform manipulation when these characteristics are present are unknown, they may represent situations with a higher perceived risk or lower perceived benefit of performing joint manipulation. Further investigation into these patient characteristics may be warranted to assess the effectiveness and safety of performing joint manipulation (versus other types of interventions) in these patient groups.

**Conclusions**

In a sample of more than 10,000 diagnostic encounters with chiropractors in Victoria, Australia and Ontario, Canada, joint manipulation was the most common therapeutic intervention for spine-related problems, whereas soft tissue therapies were more common for extremity problems. Several patient and provider characteristics were associated with therapeutic intervention selection. These data may be used to support further research on appropriate selection of therapeutic interventions for common musculoskeletal complaints.

**Declarations**

*Ethical approval and Consent to participate*

Ethics approval for COAST was provided by the University of Melbourne Human Research Ethics Committee (HREC: 0931651) and for O-COAST by the Canadian Memorial Chiropractic College (REB: 1404X03) and Queen’s University (REB: 6012853) ethics boards.

*Consent for publication*

Not applicable

*Availability of data and materials*

The datasets used and/or analysed during the current study are available from the corresponding author on reasonable request

*Competing interests*

SDF is Co-Editor-in-Chief of Chiropractic & Manual Therapies. The editorial management system automatically blinded him from the submitted manuscript, and he had no part in the editorial or peer-review process of this manuscript.

JJY is supported by Arthritis Society Canada and the Danish Foundation for Chiropractic Research and Post-graduate Education, both outside the submitted work.

JJW is funded by a Banting Postdoctoral Fellowship from the Canadian Institutes of Health Research; and reports grants from the Canadian Institutes of Health Research and Canadian Chiropractic Research Foundation (paid to university) outside the submitted work.

The other authors declare that they have no competing interests.

*Funding*

This COAST study was funded by the Chiropractors Registration Board of Victoria and the Chiropractors Association of Australia. The funding bodies provided general advice about the design of the project but had no role in the conduct of the study or in the decisions about publication of the results.

This O-COAST study was funded by the Ontario Chiropractic Association (OCA) and Queen’s University. The OCA provided general advice about the design of the project, but was not involved in the collection of data, data analysis, interpretation of data or drafting of the manuscript. The funder Queen’s University did not have any involvement in the project.

No additional funding was obtained for this study

*Author contributions*

HJJ was involved in the design of the study, performed the data synthesis and analysis, performed and agreed upon the data interpretation, and drafted, reviewed, and approved the final manuscript.

AD was involved in the design of the study, reviewed and agreed upon the data interpretation, and reviewed and approved the final manuscript.

JJW was involved in the design of the study, reviewed and agreed upon the data interpretation, and reviewed and approved the final manuscript.

JJY was involved in the design of the study, reviewed and agreed upon the data interpretation, and reviewed and approved the final manuscript.

EJR was involved in the design of the study, reviewed and agreed upon the data interpretation, and reviewed and approved the final manuscript.

CGN was involved in the design of the study, reviewed and agreed upon the data interpretation, and reviewed and approved the final manuscript.

DM was involved in the design of the study, reviewed and agreed upon the data interpretation, and reviewed and approved the final manuscript.

CKØ was involved in the design of the study, reviewed and agreed upon the data interpretation, and reviewed and approved the final manuscript.

JH was involved in the design of the study, reviewed and agreed upon the data interpretation, and reviewed and approved the final manuscript.

SM was involved in the design of the study, reviewed and agreed upon the data interpretation, and reviewed and approved the final manuscript. SM was the lead investigator for the O-COAST study.

SDF was involved in the design of the study, reviewed and agreed upon the data interpretation, and reviewed and approved the final manuscript. SDF was the lead investigator for the COAST study.

*Acknowledgements*

Not applicable

**References**

1. World Health Organisation: **WHO guidelines on basic training and safety in chiropractic.** Geneva: World Health Organisation; 2005.

2. Stochkendahl MJ, Rezai M, Torres P, Sutton D, Tuchin P, Brown R, Côté P: **The chiropractic workforce: a global review.** *Chiropractic & Manual Therapies* 2019, **27**.

3. Beliveau PJ, Wong JJ, Sutton DA, Simon NB, Bussières AE, Mior SA, French SD: **The chiropractic profession: a scoping review of utilization rates, reasons for seeking care, patient profiles, and care provided.** *Chiropractic & Manual Therapies* 2017, **25:**35.

4. Walker BF, French SD, Grant W, Green S: **Combined chiropractic interventions for low-back pain.** *Cochrane Database of Systematic Reviews* 2010, **2010:**Cd005427.

5. Sackett DL, Rosenberg WMC, Gray JAM, Haynes RB, Richardson WS: **Evidence based medicine: what it is and what it isn't.** *BMJ* 1996, **312:**71-72.

6. Vining RD, Shannon ZK, Salsbury SA, Corber L, Minkalis AL, Goertz CM: **Development of a Clinical Decision Aid for Chiropractic Management of Common Conditions Causing Low Back Pain in Veterans: Results of a Consensus Process.** *Journal of Manipulative and Physiological Therapeutics* 2019, **42:**677-693.

7. Clijsters M, Fronzoni F, Jenkins H: **Chiropractic treatment approaches for spinal musculoskeletal conditions: a cross-sectional survey.** *Chiropractic & Manual Therapies* 2014, **22:**1-10.

8. French SD, Charity MJ, Forsdike K, Gunn JM, Polus BI, Walker BF, Chondros P, Britt HC: **Chiropractic Observation and Analysis Study (COAST): providing an understanding of current chiropractic practice.** *Medical Journal of Australia* 2013, **199:**687-691.

9. Mior S, Wong J, Sutton D, Beliveau PJ, Bussières A, Hogg-Johnson S, French S: **Understanding patient profiles and characteristics of current chiropractic practice: a cross-sectional Ontario Chiropractic Observation and Analysis STudy (O-COAST).** *BMJ Open* 2019, **9:**e029851.

10. Von Elm E, Altman DG, Egger M, Pocock SJ, Gøtzsche PC, Vandenbroucke JP: **The Strengthening the Reporting of Observational Studies in Epidemiology (STROBE) statement: guidelines for reporting observational studies.** *Journal of Clinical Epidemiology* 2008, **61:**344-349.

11. Charity MJ, French SD, Forsdike K, Britt H, Polus B, Gunn J: **Extending ICPC-2 PLUS terminology to develop a classification system specific for the study of chiropractic encounters.** *Chiropractic & Manual Therapies* 2013, **21:**4.

12. **College of Chiropractors Ontario Registry data 2013-14;** In an email from M Fillery (mfillery@cmcc.ca) on September 22, 2015.

13. Lamberts H, Wood M: *ICPC, International Classification of Primary Care.* Oxford University Press, USA; 1987.

14. Dunn AS, Passmore SR: **Consultation request patterns, patient characteristics, and utilization of services within a Veterans Affairs medical center chiropractic clinic.** *Military medicine* 2008, **173:**599-603.

15. Chu EC-P, Trager RJ, Lee LY-K, Niazi IK: **A retrospective analysis of the incidence of severe adverse events among recipients of chiropractic spinal manipulative therapy.** *Scientific Reports* 2023, **13**.

16. Dudley R, Ingham B, Sowerby K, Freeston M: **The utility of case formulation in treatment decision making; the effect of experience and expertise.** *Journal of Behavior Therapy and Experimental Psychiatry* 2015, **48:**66-74.

17. Bate L, Hutchinson A, Underhill J, Maskrey N: **How clinical decisions are made.** *British Journal of Clinical Pharmacology* 2012, **74:**614-620.

18. Nuttall FQ: **Body mass index: obesity, BMI, and health: a critical review.** *Nutrition Today* 2015, **50:**117.

19. Australian Government Department of Health. **Chiropractors** 2019 [February, 2023]. Available from: <https://hwd.health.gov.au/resources/publications/factsheet-alld-chiropractors-2019.pdf>, Accessed February, 2023

20. Oliveira CB, Maher CG, Pinto RZ, Traeger AC, Lin C-WC, Chenot J-F, van Tulder M, Koes BW: **Clinical practice guidelines for the management of non-specific low back pain in primary care: an updated overview.** *European Spine Journal* 2018**:**1-13.

21. Côté P, Wong JJ, Sutton D, Shearer HM, Mior S, Randhawa K, Ameis A, Carroll LJ, Nordin M, Yu H, et al: **Management of neck pain and associated disorders: A clinical practice guideline from the Ontario Protocol for Traffic Injury Management (OPTIMa) Collaboration.** *European Spine Journal* 2016, **25:**2000-2022.

22. Lin I, Wiles L, Waller R, Goucke R, Nagree Y, Gibberd M, Straker L, Maher CG, O’Sullivan PPB: **What does best practice care for musculoskeletal pain look like? Eleven consistent recommendations from high-quality clinical practice guidelines: systematic review.** *British Journal of Sports Medicine* 2020, **54:**79-86.

23. O'Hagan ET, Cashin AG, Traeger AC, McAuley JH: **Person-centred education and advice for people with low back pain: Making the best of what we know.** *Brazilian Journal of Physical Therapy* 2023, **27:**100478.

**Tables**

Table 1: Therapeutic intervention categorisation definitions and method of data collection in COAST and O-COAST

| **Therapeutic intervention category** | **Definition** | **Data collection method** |
| --- | --- | --- |
| Manipulation | High-velocity low-amplitude (HVLA) force applied to a joint by hand, without additional instrument or table assistance. Maitland grade V | Tick-box |
| Mobilisation | Force applied to a joint by hand, without a HVLA component. Maitland grade I-IV | Tick-box |
| Drop-piece | Force applied to a joint using a drop-piece table | Tick-box |
| Instrument adjusting | Force applied to a joint using a hand-held instrument (e.g., Activator, arthrostim, TRT) | Tick-box |
| Flexion-distraction | Force applied to a joint using a flexion-distraction table | Tick-box |
| Blocks | Positioning of blocks under the pelvis to create a passive positional change | Tick-box |
| Chiropractic system | Chiropractic systems of treatment defined by the chiropractor (e.g., Network, sacrooccipital technique, neuroemotional technique) | Free-text |
| Soft tissue techniques | Treatment to decrease tension of lengthen soft tissue structures (e.g., massage, trigger point release, passive stretches) | Tick-box |
| Advice/Education | Provision of advice or education to the patient | Free-text |
| Exercise prescription | Provision of exercises to the patient, either during the visit or to perform at home | Free-text |
| Modalities | Use of additional passive treatment modalities, not described above (e.g., heat/ice, ultrasound, laser, shockwave) | Free-text COAST; Tick-box O-COAST |
| Acupuncture | Use of acupuncture or dry-needling | Free-text COAST; Tick-box O-COAST |
| Supportive devices | Provision of supportive devices (e.g., orthotics, braces, taping) | Free-text |

Table 2: Characteristics of patients, providers, and diagnostic groupings across all diagnostic encounters*, overall and stratified by country of practice

| **Variables** | **Overall (N=10731)** | **Australia (N=6123, 57%)** | **Canada (N=4608, 43%)** |
| --- | --- | --- | --- |
| Patient variables |  |  |  |
| Age (Mean, SD) | 43.7 (20.7) | 42.9 (19.7) | 44.8 (22.0) |
| Sex (Female; n/N, %) | 6034/10441 (57.8) | 3411/5996 (56.9) | 2623/4445 (59.0) |
| Comorbidity reported (n/N, %) | 3036/10731 (28.3) | 1647/6123 (26.9) | 1389/4608 (30.1) |
| BMI (n/N, %) |  |  |  |
| Underweight   (BMI < 18.5) | 500/10076 (5.0) | 367/5861 (6.3) | 133/4215 (3.2) |
| Normal weight   (BMI 18.5 - < 25) | 3717/10076 (36.9) | 2117/5861 (36.1) | 1600/4215 (38.0) |
| Overweight   (BMI 25 - < 30) | 3470/10076 (34.4) | 1989/5861 (33.9) | 1481/4215 (35.1) |
| Obese   (BMI ≥30) | 2389/10076 (23.7) | 1388/5861 (23.7) | 1001/4215 (23.7) |
| New patient (n/N, %) | 583/8912 (6.5) | 311/4628 (6.7) | 272/4284 (6.3) |
| New complaint  (n/N, %) | 3017/9039 (33.4) | 1918/5108 (37.5) | 1099/3931 (28.0) |
| Work related complaint (n/N, %) | 1956/8856 (22.1) | 1335/5068 (26.3) | 621/3788 (16.4) |
| Chiropractor variables |  |  |  |
| Age (Mean, SD) | 43.7 (10.2) | 42.5 (9.3) | 45.4 (11.1) |
| Years in practice  (n/N, %) |  |  |  |
| ≤5 years | 1376/10731 (12.8) | 601/6123 (9.8) | 775/4608 (16.8) |
| >5 years | 9355/10731 (87.2) | 5522/6123 (90.2) | 3833/4608 (83.2) |
| Average patient visits per week (Mean, SD) | 97.3 (62.0) | 85.0 (43.1) | 113.8 (77.5) |
| Sex (Female; n/N, %) | 3282/10731 (30.6) | 1757/6123 (28.7) | 1525/4608 (33.1) |
| Involved in teaching (n/N, %) | 1506/10532 (14.3) | 828/6123 (13.5) | 678/4409 (15.4) |
| Diagnostic grouping (n/N, %) |  |  |  |
| Musculoskeletal - Back | 6285/10731 (58.6) | 3558/6123 (58.1) | 2727/4608 (59.2) |
| Musculoskeletal - Neck | 1391/10731 (13.0) | 682/6123 (11.1) | 709/4608 (15.4) |
| Musculoskeletal - Head/Jaw | 293/10731 (2.7) | 220/6123 (3.6) | 73/4608 (1.6) |
| Musculoskeletal - Extremity | 985/10731 (9.2) | 402/6123 (6.6) | 583/4608 (12.7) |
| Musculoskeletal - Non Region-Specific | 1187/10731 (11.1) | 783/6123 (12.8) | 404/4608 (8.8) |
| Non-musculoskeletal problem | 315/10731 (2.9) | 242/6123 (4.0) | 73/4608 (1.6) |
| Health maintenance /Prevention | 275/10731 (2.6) | 236/6123 (3.9) | 39/4608 (0.8) |

*The table is presented by diagnostic encounter not per patient/provider. Each patient may have had multiple visits with a chiropractor, and at each visit may have received up to three diagnoses. Patients may have received the same diagnosis at multiple visits.

**Figures**

Figure 1: Frequency of therapeutic interventions selected across all diagnostic encounters and for each diagnostic grouping*

*Presented as percentage of diagnostic encounters where the therapeutic intervention was selected, with 95%CI

Figure 2: Frequency of therapeutic interventions selected across all diagnostic encounters, stratified by country of practice*

*Presented as percentage of diagnostic encounters where the therapeutic intervention was selected with 95%CI

Figure 3: Univariable association between patient and provider variables and the use of manipulation, mobilisation, or other chiropractic techniques across all diagnostic encounters*

*BMI reference category is normal weight

Figure 4: Univariable association between patient and provider variables and the use of soft tissue techniques, advice/education, exercise prescription, or ancillary care across all diagnostic encounters*

*****BMI reference category is normal weight
